# Norsesquiterpenes from *Lolium perenne* and Their Replacement Control of an Invasive Plant, *Ageratina adenophora*, Through Allelopathy

**DOI:** 10.3390/molecules30112384

**Published:** 2025-05-29

**Authors:** Wenbo Shi, Tong An, Xiaomin Yang, Youlin Li, Amanula Yimingniyazi, Zhixiang Liu, Yulong Feng

**Affiliations:** 1 College of Bioscience and Biotechnology, Shenyang Agricultural University, Shenyang 110866, China; swb111@126.com (W.S.); m18602463916@163.com (T.A.); 19355636367@163.com (X.Y.); 2 College of Resources and Environment, Yunnan Agricultural University, Kunming 650500, China; panzer416@163.com; 3 Xinjiang Key Laboratory for Ecological Adaptation and Evolution of Extreme Environment Biology, College of Life Sciences, Xinjiang Agricultural University, Urumqi 830052, China; amanula.y@xjau.edu.cn

**Keywords:** *Ageratina adenophora*, allelopathy, *Lolium perenne*, norsesquiterpenes, replacement control

## Abstract

*Lolium perenne* (Poaceae), a perennial forage, has high economic and nutritional value. It is often used as a replacement control for some invasive plants, as it has achieved good ecological and economic effects. However, its control effects, allelochemicals, allelopathic effects, release pathways, and contents are still unclear in the process of *L. perenne* replacement control of an invasive plant, *Ageratina adenophora* (Asteraceae). Therefore, it is necessary to reveal the mechanism of *L. perenne* replacement control of *A. adenophora* from the perspective of allelopathy. In this study, *L. perenne* could effectively inhibit the growth of *A. adenophora* in the competition assay. In addition, seven norsesquiterpenes (**1**–**7**) were isolated and identified from the whole plant of *L. perenne*, and most of the compounds exhibited potent allelopathic effects on the growth of *A. adenophora* and one model plant (*Lactuca sativa*, Asteraceae). Moreover, some active compounds were released into the environment through root secretion and rainwater leaching, and their contents were determined by UPLC-MS/MS (Ultra Performance Liquid Chromatography Tandem Mass Spectrometry). Our results elucidated the allelopathic mechanism of *L. perenne*’s replacement control, *A. adenophora*, and provided a theoretical basis for the development of norsesquiterpenes from *L. perenne*.

## 1. Introduction

In recent decades, invasive plants have seriously harmed the sustainable development of agriculture, forestry, animal husbandry, and fishery, causing a serious threat to ecological security and human health in many countries around the world. People have been trying to prevent the invasion of alien plants by various means, but the types and numbers of invasive plants have not shown any signs of disappearing [1]. On the contrary, as time went on, invasive plants showed an expanding trend, and the scope and area of their harm also increased [2]. Therefore, preventing the spread of invasive plants and realizing sustainable control of existing invasive plants have become major demands to ensure the coordinated development of social progress and nature. In the past, the comprehensive control methods of invasive plants mainly focused on artificial control, chemical control, and natural enemy control. Although these methods have achieved some level of success, there many shortcomings remain, such as the time and effort required, environmental pollution, and secondary invasion [3,4]. Therefore, it is urgent to find an economical, environmentally friendly, effective, and widely applied control method.

Replacement control is an important ecological control method for invasive plants. It is a natural succession process of replacing target invasive plants with economically or ecologically valuable native plants [5]. The advantage of replacement control is that it can control invasive plants for a long time. It has played an important role in conserving water and soil, improving soil environment and protecting community diversity. In addition, suitable alternative plants can also provide certain economic value. Nowadays, replacement control has been widely used in the control of invasive plants [6,7].

In recent years, many scholars have conducted a lot of research on the mechanisms of replacement control, among which allelopathy is a driving factor in the successful replacement control of invasive plants [8]. Allelopathy refers to the effect that plants have on the growth of other plants by releasing specific chemicals into the environment through appropriate pathways (root secretion), and the effect is mostly the inhibition of invasive species [9,10]. Similarly, the alternative plant could gain a competitive advantage and promote it as a dominant single population by producing some allelochemicals that negatively affect the growth of invasive plants. Allelochemicals are naturally produced by competition between plants. Compared with traditional chemical pesticides, they can effectively prevent the resistance of invasive plants and reduce the pollution of chemical herbicides to the environment [11,12]. Therefore, using allelopathy as replacement control of invasive plants is an effective measure with broad prospects and sustainable development.

*Lolium perenne* L. is a perennial grass of the Poaceae family, which is widely distributed in Asia, Europe, and Africa [13]. It is a high-quality grazing grass characterized by fast growth, many tillers, and high digestible matter yields [14]. In addition, it has high nutritional value and is rich in protein, minerals, and vitamins [15]. In addition, it has high economic value and is commonly used in urban greening and golf courses [16]. Based on the above characteristics of *L. perenne*, it is often used as a replacement control of some invasive plants and has achieved good ecological and economic outcomes [17].

*Ageratina adenophora* (Spreng.), according to R. M. King and H. Rob, is a perennial malignant weed of Asteraceae. It originated from Mexico in Central America and was introduced to Europe, Australia, and Asia as an ornamental plant in the 19th century. Now, it is widely distributed in many countries and regions in the tropics and subtropics [18]. In China, *A. adenophora* first invaded Yunnan Province in the 1940s from Myanmar. Now, it has spread to Guizhou, Sichuan, Guangxi, Chongqing, and other places, becoming one of the main invasive plants in southwest China [19]. Because of its vigorous vitality, strong fecundity, and wide adaptability, *A. adenophora* has invaded many habitats, such as farmland, gardens, and forests. It repels the growth of other plants in invasion areas to form a dominant single population. In addition, its branches, seeds, and pollen are toxic, causing allergic asthma in livestock and humans, and can even lead to death by poisoning [20].

Currently, the comprehensive control methods of *A. adenophora* mainly focus on artificial control, chemical control, and natural enemy control [17]. Although these methods have achieved some level of success, there are remain shortcomings, such as time and effort required, environmental pollution, and secondary invasion. Replacement control could promote the gradual restoration of natural ecosystem and is receiving more and more attention [6]. Many studies have shown that *L. perenne* could competitively inhibit the growth of *A. adenophora*, suggesting that *L. perenne* has good potential as a replacement control of *A. adenophora* [17,21,22]. Our previous experiment showed that the water extract of *L. perenne* had significant inhibitory effects on the root length, stem length, and seed germination of *A. adenophora* (Figure S16), indicating that allelopathy of *L. perenne* could be a driving factor in its successful replacement control of *A. adenophora*.

However, the control effects, allelochemicals, allelopathic effects, release pathways, and contents are still unclear in the process *L. perenne*’s replacement control of *A. adenophora*. Therefore, it is necessary to reveal the mechanism of *L. perenne*’s replacement control on *A. adenophora* from the perspective of allelopathy. In this study, to investigate the replacement control effects of *L. perenne* on *A. adenophora*, we conducted a growth competition assay between the two plants. In addition, to clarify the allelochemicals of *L. perenne*, we isolated and identified the secondary metabolites from the whole plant of *L. perenne*. The allelopathic effects of allelochemicals on *A. adenophora* and one model plant (*Lactuca sativa*) were also investigated. Moreover, the release pathways and contents of allelochemicals were analyzed using UPLC-MS/MS.

## 2. Results and Discussion

### 2.1. Growth Competition Assay

**Figure 1:**
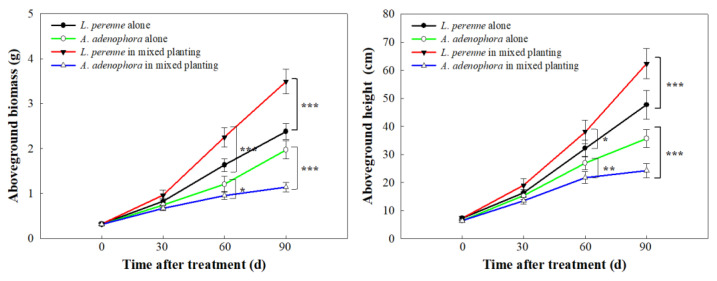
Growth performance of *L. perenne* and *A. adenophora* in monoculture and mixed culture. Different asterisks indicat significant differences between different cultivation treatments (* *p* < 0.05; ** *p* < 0.01; *** *p* < 0.001).

In the growth competition assay, the aboveground height and biomass are two important indicators, which are directly related to whether one plant could occupy the ecological niche quickly and inhibit the growth of another plant [23]. As shown in Figure 1, the aboveground height and biomass of *L. perenne* and *A. adenophora* showed a gradually increasing trend with the increase in time. From 0 to 30 d, the aboveground height and biomass of the two plants under different cultivation treatments had little difference. From 60 to 90 d, compared with alone planting, the aboveground height and biomass of *L. perenne* in mixed planting were significantly increased, especially at 90 d (62.34 ± 5.36 cm of aboveground height and 3.49 ± 0.27 g of aboveground biomass, respectively). This phenomenon showed that the growth of *L. perenne* was not affected by *A. adenophora*, and *L. perenne* had good ecological adaptability and could establish a dominant population. Previous studies have shown that *L. perenne* could competitively inhibit the growth of *A. adenophora* and has good potential for replacement control of *A. adenophora* [17,21,22], which further confirmed our results. On the other hand, compared with alone planting, the aboveground height and biomass of *A. adenophora* were significantly decreased, especially at 90 d (24.26 ± 2.57 cm of aboveground height and 1.14 ± 0.10 g of aboveground biomass, respectively). This phenomenon showed that *L. perenne* could competitively inhibit the growth of *A. adenophora* and that *L. perenne* has good potential as a replacement control of *A. adenophora*. From the perspective of allelopathy, we speculated that *L. perenne* could gain a competitive advantage by releasing some specific allelochemicals that negatively affect the growth of *A. adenophora*.

### 2.2. Structural Identification

**Figure 2:**
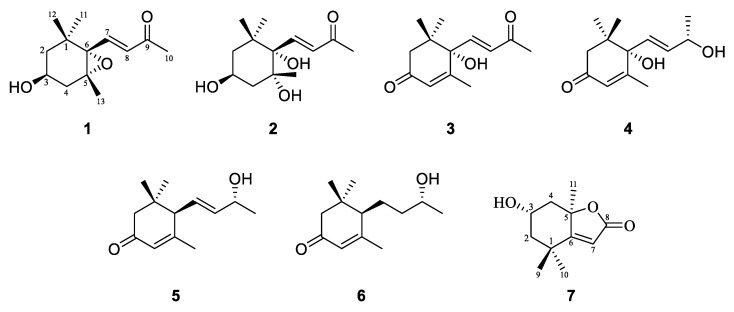
Structures of compounds **1**–**7**.

As shown in Figure 2, the compounds (**1**–**7**) were identified as 6-Epoxy-3-hydroxy-7-megastigmen-9-one (**1**) [24], (3*S*, 5*R*, 6*S*, 7*E*)-3, 5, 6-Trihydroxy-7-megastigmen-9-one (**2**) [25], (+)-Dehydrovomifoliol (**3**) [25], Corchoionol C (**4**) [26,27], (6*R*, 7E, 9*R*)-9-Hydroxy-4, 7-megastigmadien-3-one (**5**) [25], (6*R*, 9*R*)-9-Hydroxy-4-megastigmen-3-one (**6**) [25], and Loliolide (**7**) [28,29], respectively, by comparison of their NMR (nuclear magnetic resonance) and optical rotation data with those reported in the literature. The NMR data of these compounds are listed in the Supporting Information (Figures S1–S14). Based on structural skeleton analyses, they all belonged to norsesquiterpenes. Previous studies on the chemical compositions of *L. perenne* have been limited; only some indole diterpenes and epoxyjanthitrems have been isolated from *L. perenne* infected with some endophytic fungi [30,31]. Therefore, the isolation and identification of these norsesquiterpenes not only enriched the chemical compositions of *L. perenne* but also provided a theoretical basis for the development and utilization of the chemical resources of this plant.

### 2.3. Allelopathic Assay of Compounds

**Figure 3:**
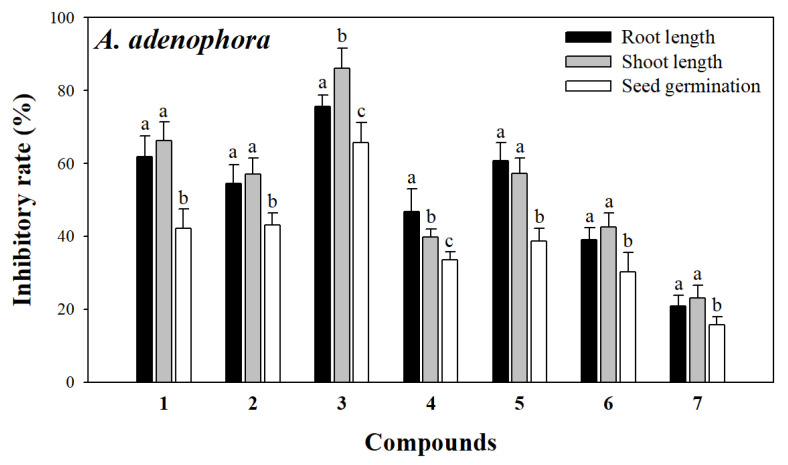
Allelopathic effects of compounds **1**–**7** on root length, stem length, and seed germination of *A. adenophora*. The different letters (a, b, c) indicate significant differences between different growth indicators for each compound (*p* < 0.05).

**Figure 4:**
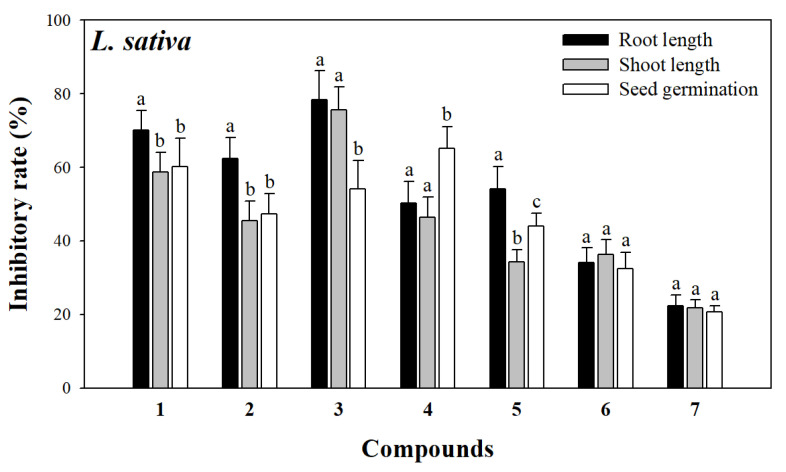
Allelopathic effects of compounds **1**–**7** on root length, stem length, and seed germination of *L. sativa*. The different letters (a, b, c) indicate significant differences between different growth indicators for each compound (*p* < 0.05).

Studies have shown that norsesquiterpenes are important small-molecule compounds in allelopathy and could significantly inhibit plant growth [32,33]. Similarly, these norsesquiterpenes may be potential allelochemicals of *L. perenne*. As shown in Figure 3 and Figure 4, all of the compounds exhibited different levels of allelopathic effects on root length, stem length, and seed germination of *A. adenophora* and *L. sativa*. Among them, compounds **1**–**6** exhibited moderate to strong allelopathic effects at 200 μg mL^−1^ on *A. adenophora* with inhibitory rates of root length, stem length, and seed germination ranging from 39.01 ± 3.29 to 77.59 ± 3.17%, 39.77 ± 2.25 to 86.22 ± 5.48%, and 30.16 ± 5.33 to 65.80 ± 5.39%, and on *L. sativa* with inhibitory rates of root length, stem length, and seed germination ranging from 34.11 ± 4.02 to 78.42 ± 7.91%, 36.25 ± 4.06 to 75.66 ± 6.27%, and 32.50 ± 4.29 to 65.16 ± 5.94%, respectively. Compound **7** exhibited slight allelopathic effects at 200 μg mL^−1^ on *A. adenophora* with inhibitory rates of 20.94 ± 2.84% on root length, 23.16 ± 3.40% on stem length, and 15.78 ± 2.25% on seed germination, and on *L. sativa* with inhibitory rates of 22.26 ± 2.94% on root length, 21.77 ± 2.28% on stem length, and 20.67 ± 1.61% on seed germination, respectively. In addition, compounds **1**–**7** inhibited the root length and stem length of *A. adenophora* more significantly than seed germination, indicating that these norsesquiterpenes occupied ecological niches and gained competitive advantage by inhibiting the root length and stem length of *A. adenophora*.

Notably, compound **3** showed remarkable allelopathic effects on *A. adenophora* (75.59 ± 3.17% on root length, 86.22 ± 5.48% on stem length, and 65.80 ± 5.39% on seed germination, respectively) and on *A. adenophora* (78.42 ± 7.91% on root length, 75.66 ± 6.27% on stem length, and 54.18 ± 7.72% on seed germination, respectively) compared to other compounds. Based on structure-activity analyses of compounds **1**–**7**, we speculated that norsesquiterpenes with carbonyl groups at C-3 and C-9 may have potent allelopathic effects. Previous studies have shown that compound **3** has significant allelopathic effects on many plants, such as *Oenanthe javanica* [32], *Amaranthus retrflexus*, *Poa annua* [28], and *Lolium multiflorum* [34], which further confirmed our results. Meanwhile, it is also the first time we found that compound **3** has a significant inhibitory effect on the growth of one invasive plant (*A. adenophora*) and one model plant (*L. sativa*). Compound **3** with potent allelopathic effects is expected to be developed into a plant-derived herbicide.

### 2.4. UPLC-MS/MS Analyses

**Figure 5:**
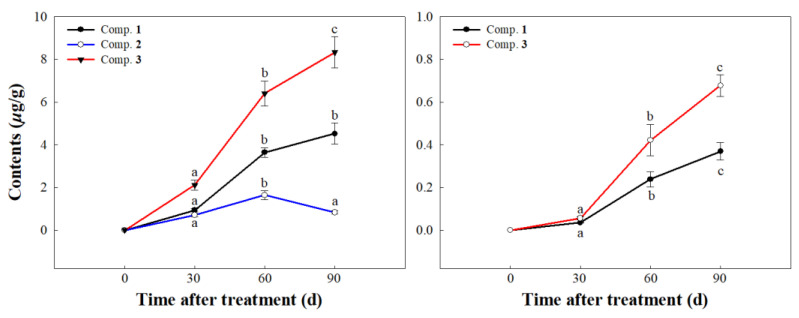
UPLC-MS/MS analyses of compounds in the root secretion and rainwater leaching of *L. perenne*. The different letters (a, b, c) indicate significant differences in contents between different times for each compound (*p* < 0.05).

Root secretion and rainwater leaching are two essential carriers for material circulation, energy exchange, and information transfer between plants and soil, as well as the important release pathways of allelochemicals [35,36]. Because of the low allelopathic effects of compound **7** and as the small amounts of compounds **4** and **6** are isolated, compounds **1**, **2**, **3**, and **5** in the root secretion and the rainwater leaching of *L. perenne* were selected for further UPLC-MS/MS analyses. As shown in Figure 5, the contents of compounds **1** and **3** in the root secretion and the rainwater leaching showed a gradually increasing trend with the increase in time. At 90 d, the concentrations of compounds **1** and **3** in the root secretion and the rainwater leaching were determined as 4.52 ± 0.49 and 8.32 ± 0.72 μg/g and 0.36 ± 0.04 and 0.67 ± 0.05 μg/g, respectively. The reason for the low contents of compounds in the rainwater leaching may be due to the short rinse time of distilled water. Combined with the allelopathic assay, the results indicated that the active compounds **1** and **3** were released into the environment, mainly through root secretion, to negatively affect the growth of *A. adenophora*. In addition, the content of compound **2** in the root secretion showed a trend of first increasing and then decreasing with the increase in time. We speculated that compound **2** might be degraded by some microorganisms in the soil, resulting in a decrease in its content. Notably, the content of compound **5** did not change with the increase in time. It indicated that compound **5** was not released into the environment through root secretion and rainwater leaching but probably through other pathways, such as residue degradation or gas volatilization. Further research on the release pathway of compound **5** is needed to confirm this.

## 3. Materials and Methods

### 3.1. Plant Material

The seeds of *A. adenophora* were collected from Kunming, Yunnan Province, China (102°40′56″ E, 25°1′36″ N) in April 2024. The seeds of *L. sativa* were collected from Shandong Shouhe Seed Industry Co., Ltd. (Weifang, China) in March 2024. The seeds and whole plants of *L. perenne* were collected from Kunming, Yunnan Province, China (102°45′29″ E, 25°8′46″ N) in June 2024. The seeds and plants of *L. perenne*, *A. adenophorae*, and *L. sativa* were identified by Prof. Mingchao Liu (Shenyang Agricultural University).

### 3.2. Growth Competition Assay

The test of replacement control was carried out in the experimental base of Yunnan Agricultural University (102°45′54″ E, 25°8′25″ N). It is 1891 m above sea level and has an average annual temperature of 15 °C. The climate is subtropical monsoon. The average annual precipitation is 943 mm and evaporation is 1461 mm. The soil is mountainous red soil.

Firstly, the seeds of *L. perenne* and *A. adenophora* were sown in fields covered with mulch at the beginning of July 2024, respectively. After cultivation, the average plant heights of *L. perenne* and *A. adenophora* were 7.21 ± 0.73 cm and 6.36 ± 0.54 cm, respectively (n = 20). Secondly, we selected a flat and consistent plot (3 m × 3 m) and removed all weeds from the plot. Under the condition of the same total plant density per unit area, the three treatments were *L. perenne* alone, *A. adenophora* alone, and *L. perenne* and *A. adenophora* 1:1 mixed, respectively. Each treatment was performed with five replicates. Then, the two plants were transplanted into the corresponding plot according to different treatment methods. The planting density was 100 plants per square meter, and the spacing of plants was 10 cm. Different plants were cross-planted with each other. During the whole experiment, other weeds in the plot were pulled out manually. Finally, 20 plants of *L. perenne* and *A. adenophora* were randomly selected in each plot, respectively, and the aboveground heights of individual plants were measured every 30 days for a total of three times. Subsequently, the aboveground part of each plant was dried in an oven at 80 °C for 48 h to obtain the aboveground biomass. The data of growth competition assay were presented as the mean ± SD (Standard Deviation) of the replicates. One-way ANOVA (analysis of variance) was used for statistical comparisons between the groups.

### 3.3. Extraction and Isolation

The whole plant of *L. perenne* (dry weight 10.0 kg) was cut into 3 cm pieces manually and extracted with distilled water (120 L, 8 days, at room temperature) three times. The water extract was combined and filtered, and the filtrate was concentrated by a rotary evaporator (10 L, RE-5210A, Shanghai Yarong, China). The concentrated extract (32 g) was subjected to macroporous resin chromatography (150 × 12 cm, D101, Cangzhou Baoen, China) eluted with CH_3_OH:H_2_O (0:100, 30:70, 60:40, 100:0, *v*/*v*) to obtain four subfractions (Fr. A–Fr. D). Fr. C (3.7 g) was subjected to silica chromatography (60 × 5.5 cm, 200–300 mesh, Qingdao Marine, China) eluted with CH_2_Cl_2_:CH_3_OH (98:2, 95:5, 90:10, 80:20, 70:30, *v*/*v*) to obtain five subfractions (Fr. C-1–Fr. C-5). Fr. C-2 (337 mg) was subjected to gel chromatography (150 × 2 cm, Sephadex LH-20, GE Healthcare, Sweden) eluted with CH_3_OH:H_2_O (90:10, *v*/*v*) to obtain three subfractions (Fr. C-2-1–Fr. C-2-3). Fr. C-2-1 (94 mg) was isolated by HPLC (High Performance Liquid Chromatography) (4.0 mL/min, 1260 II, Agilent, Santa Clara, CA, USA) equipped with a preparative column (150 × 4.6 mm, 5 μm, XDB-C18, Agilent, USA) and eluted with CH_3_CN:H_2_O (28:72, *v*/*v*) to obtain compounds **3** (8.2 mg, t*_R_* = 27.1 min), **5** (5.4 mg, t*_R_* = 35.3 min), and **6** (2.8 mg, t*_R_* = 41.8 min), respectively. Fr. C-2-2 (62 mg) was isolated by preparative HPLC (4.0 mL/min, 1260 II, Agilent, USA) eluted with CH_3_CN:H_2_O (30:70, *v*/*v*) to obtain compound **7** (16.5 mg, t*_R_* = 57.6 min). Fr. C-3 (220 mg) was subjected to ODS (Octadecylsilyl) chromatography (40 × 2 cm, 50 μm, YMC, Kyoto, Japan) eluted with CH_3_OH:H_2_O (10:90, 30:70, 60:30, 90:10, *v*/*v*) to obtain five subfractions (Fr. C-3-1–Fr. C-3-5). Fr. C-3-2 (48 mg) was isolated by preparative HPLC (4.0 mL/min, 1260 II, Agilent, USA) eluted with CH_3_CN:H_2_O (20:80, *v*/*v*) to obtain compounds **2** (5.7 mg, t*_R_* = 30.5 min) and **4** (2.6 mg, t*_R_* = 37.6 min), respectively. Fr. C-3-3 (45 mg) was isolated by preparative HPLC (4.0 mL/min, 1260 II, Agilent, USA) eluted with CH_3_CN:H_2_O (20:80, *v*/*v*) to obtain compound **1** (8.5 mg, t*_R_* = 42.2 min).

### 3.4. Structural Identification

The structures of compounds (**1**–**7**) were determined by NMR spectroscopy (AV-600, Bruker, Ingelfingen, Germany) and optical rotation (241, PerkinElmer, Santa Clara, CA, USA). The solvent used in NMR and optical rotation was CD_3_OD (Macklin, Shanghai, China). The NMR was operated at 600 MHz (^1^H) and 150 MHz (^13^C) and equipped with an ultra-low temperature probe.

Compound **1**, colorless oil; [α${]}_{D}^{20}$: −60.12 (c 0.10, CH_3_OH); ^1^H-NMR (CD_3_OD, 600 MHz): *δ* 7.18 (1H, d, *J* = 15.2 Hz, H-7), 6.19 (1H, d, *J* = 15.2 Hz, H-8), 3.77 (1H, m, H-3), 2.32 (1H, m, H-4a), 2.31 (3H, s, H-10), 1.68 (1H, dd, *J* = 14.7, 9.2 Hz, H-4b), 1.59 (1H, dd, *J* = 12.3, 4.2 Hz, H-2a), 1.29 (1H, dd, *J* = 12.3, 10.1 Hz, H-2b), 1.25 (3H, s, H-13), 1.19 (3H, s, H-12), 0.97 (3H, s, H-11); ^13^C-NMR (CD_3_OD, 150 MHz): *δ* 200.2 (C-9), 145.4 (C-7), 133.8 (C-8), 70.8 (C-6), 68.7 (C-5), 64.3 (C-3), 47.6 (C-4), 41.3 (C-2), 36.0 (C-1), 29.7 (C-12), 27.4 (C-10), 25.1 (C-11), 20.0 (C-13).

Compound **2**, colorless amorphous powder; [α${]}_{D}^{20}$: −192.60 (c 0.10, CH_3_OH); ^1^H-NMR (CD_3_OD, 600 MHz): δ 7.18 (1H, d, *J* = 15.5 Hz, H-7), 6.19 (1H, d, *J* = 15.5 Hz, H-8), 3.79 (1H, m, H-3), 2.32 (1H, m, H-4a), 2.29 (3H, s, H-10), 1.68 (1H, dd, *J* = 14.5, 9.2 Hz, H-4b), 1.59 (1H, dd, *J* = 12.8, 3.5 Hz, H-2a), 1.29 (1H, dd, *J* = 12.8, 10.7 Hz, H-2b), 1.19 (3H, s, H-12), 1.18 (3H, s, H-13), 0.96 (3H, s, H-11); ^13^C-NMR (CD_3_OD, 150 MHz): *δ* 200.2 (C-9), 145.4 (C-7), 133.8 (C-8), 70.8 (C-6), 68.7 (C-5), 64.3 (C-3), 47.6 (C-2), 41.3 (C-4), 36.0 (C-1), 29.7 (C-12), 27.4 (C-10), 25.1 (C-11), 20.0 (C-13).

Compound **3**, colorless amorphous powder; [α${]}_{D}^{20}$: +223.85 (c 0.10, CH_3_OH); ^1^H-NMR (CD_3_OD, 600 MHz): *δ* 7.01 (1H, d, *J* = 15.5 Hz, H-7), 6.45 (1H, d, *J* = 15.5 Hz, H-8), 5.93 (1H, br s, H-4), 2.61 (1H, d, *J* = 17.0 Hz, H-2a), 2.31 (3H, s, H-10), 2.29 (1H, d, *J* = 17.0 Hz, H-2b), 1.90 (3H, d, *J* = 1.2 Hz, H-13), 1.06 (3H, s, H-11), 1.02 (3H, s, H-12); ^13^C-NMR (CD_3_OD, 150 MHz): *δ* 200.8 (C-9), 200.4 (C-3), 164.6 (C-5), 148.4 (C-7), 131.7 (C-8), 128.0 (C-4), 79.9 (C-6), 50.5 (C-2), 42.6 (C-1), 24.7 (C-10), 24.7 (C-12), 23.5 (C-11), 19.1 (C-13).

Compound **4**, colorless oil; [α${]}_{D}^{20}$: +176.24 (c 0.10, CH_3_OH); ^1^H-NMR (CD_3_OD, 600 MHz): *δ* 5.88 (1H, s, H-4), 5.79 (1H, br s, H-7), 5.79 (1H, br s, H-8), 4.33 (1H, m, H-9), 2.53 (1H, d, *J* = 17.0 Hz, H-2a), 2.17 (1H, d, *J* = 17.0 Hz, H-2b), 1.93 (3H, s, H-11), 1.25 (3H, d, *J* = 6.4 Hz, H-10), 1.04 (3H, s, H-13), 1.01 (3H, s, H-12); ^13^C-NMR (CD_3_OD, 150 MHz): *δ* 201.2 (C-3), 167.5 (C-5), 136.9 (C-8), 130.1 (C-7), 127.1 (C-4), 79.9 (C-6), 68.7 (C-9), 50.7 (C-2), 42.4 (C-1), 24.4 (C-13), 23.8 (C-12), 23.4 (C-10), 19.5 (C-11).

Compound **5**, colorless oil; [α${]}_{D}^{20}$: +155.30 (c 0.10, CH_3_OH); ^1^H-NMR (CD_3_OD, 600 MHz): *δ* 5.89 (1H, s, H-4), 5.71 (1H, dd, *J* = 15.0, 6.0 Hz, H-8), 5.60 (1H, dd, *J* = 15.0, 9.1 Hz, H-7), 4.28 (1H, m, H-9), 2.67 (1H, d, *J* = 9.1 Hz, H-6), 2.41 (1H, d, *J* = 16.8 Hz, H-2a), 2.07 (1H, d, *J* = 16.8 Hz, H-2b), 1.94 (3H, s, H-13), 1.23 (3H, d, *J* = 6.0 Hz, H-10), 1.03 (3H, s, H-12), 1.00 (3H, s, H-11); ^13^C-NMR (100 MHz, CD_3_OD): *δ* 202.0 (C-3), 166.0 (C-5), 140.2 (C-8), 127.1 (C-7), 126.1 (C-4), 68.7 (C-9), 56.6 (C-6), 48.3 (C-2), 37.1 (C-1), 28.0 (C-12), 27.2 (C-11), 23.7 (C-10), 23.7 (C-13).

Compound **6**, colorless oil; [α${]}_{D}^{20}$: −221.52 (c 0.10, CH_3_OH); ^1^H-NMR (CD_3_OD, 600 MHz): *δ* 5.88 (1H, s, H-4), 3.69 (1H, m, H-9), 2.48 (1H, d, *J* = 17.2 Hz, H-2a), 2.03 (3H, s, H-13), 2.01 (1H, d, *J* = 17.2 Hz, H-2b), 1.98 (1H, m, H-6), 1.51 (2H, m, H-8), 1.43 (2H, m, H-7), 1.16 (3H, d, *J* = 6.4 Hz, H-10), 1.08 (3H, s, H-11), 1.01 (3H, s, H-12); ^13^C-NMR (100 MHz, CD_3_OD): *δ* 202.00 (C-3), 169.5 (C-5), 125.2 (C-4), 68.50 (C-9), 52.3 (C-6), 47.9 (C-2), 39.7 (C-8), 37.3 (C-1), 29.0 (C-12), 27.4 (C-11), 27.2 (C-7), 24.8 (C-13), 23.6 (C-10).

Compound **7**, colorless crystal; [α${]}_{D}^{20}$: −95.60 (c 0.10, CH_3_OH); ^1^H-NMR (CD_3_OD, 600 MHz): *δ* 5.75 (1H, s, H-7), 4.22 (1H, m, H-3), 2.44 (1H, dt, *J* = 13.6, 2.2 Hz, H-4a), 2.01 (1H, dt, *J* = 14.2, 3.4 Hz, H-2a), 1.76 (3H, s, H-11), 1.74 (1H, dd, *J* = 13.6, 3.8 Hz, H-4b), 1.55 (1H, dd, *J* = 14.2, 3.4 Hz, H-2b), 1.47 (3H, s, H-10), 1.28 (3H, s, H-9); ^13^C-NMR (100 MHz, CD_3_OD): *δ* 185.7 (C-6), 174.4 (C-8), 113.3 (C-7), 88.9 (C-5), 67.2 (C-3), 47.9 (C-2), 46.4 (C-4), 37.1 (C-1), 31.0 (C-11), 27.4 (C-10), 26.9 (C-9).

### 3.5. Allelopathic Assay

Firstly, the seeds of *A. adenophora* and *L. sativa* were sterilized with 0.1% NaClO and washed with distilled water three times, respectively. Then, thirty seeds were evenly placed in a Petri dish (9 cm) covered with two layers of filter paper. Then, 2.5 mL of the solution containing 200 µg/mL of the compound or 5 mg/mL of the extract was moved into each Petri dish, respectively. The same volume of aqueous solution was used as a blank control. Each treatment was performed with five replicates. Subsequently, the Petri dishes were cultured in an illumination incubator (25 °C, with daylight and darkness both 12 h). The same volume of distilled water was added regularly every day to keep the filter paper moist. Finally, root length, stem length, and the number of germinated seeds of *A. adenophora* and *L. sativa* were measured after 7 days, respectively. The inhibitory rate (%) was calculated as (C-T)/C × 100 (C, average root length or stem length or the number of germinated seeds of blank control; T, average root length or stem length or the number of germinated seeds of treatment). The data of the allelopathic assay were presented as the mean ± SD of the replicates. One-way ANOVA was used for statistical comparisons between groups.

### 3.6. Collection of Root Secretion and Rainwater Leaching

After each growth index test, 20 plants of *L. perenne* were randomly selected in each plot. The rhizosphere soil (10 g) of *L. perenne* was collected from around 5–10 mm around the root in the plots of the growth competition assay. The plant residues, roots, and other impurities from the soil samples were carefully removed. Then, the soil samples were crushed, sifted, and extracted ultrasonically with 500 mL of CH_3_OH for 20 min. The extracted liquid was concentrated under vacuum to obtain the root secretion. The aerial part of *L. perenne* was washed with distilled water (20 L) for 10 min. The rinses were then collected and concentrated under vacuum to obtain the rainwater leaching.

### 3.7. UPLC-MS/MS Analyses

The qualitative and quantitative analyses of allelochemicals in the root secretion and rainwater leaching of *A. adenophora* were conducted using UPLC-MS/MS (6545 LC/Q-TOF, Agilent, USA) equipped with an analytical column (50 × 2.1 mm, 1.8 μm, SB-C18, Agilent, USA). The root secretion and rainwater leaching were filtered by a millipore filter (0.22 μm). UPLC-MS/MS was performed using the following parameters: flow rate, 0.5 mL/min; injection volume, 2 μL; column temperature of 30 °C; ionization mode, positive; data acquisition mode, IDA; gas temperature, 280 °C; gas flow, 8 L/ min; nebulizer, 35 psi; skimmer, 65 V; collision energy, 20 eV. H_2_O containing 0.2% acetic acid (A) and CH_3_CN (B) were used as the mobile phases of UPLC-MS/MS, and the elution method was set as 0−5 min, 20% B; 5−15 min, 20−70% B; and 15−20 min, 95% B. The allelochemicals in the root secretion and rainwater leaching were identified by comparing the retention time and mass spectrum of compounds **1**–**7**. The identified allelochemicals were quantified by an external standard method. The standard curves were established with the different concentrations (200, 100, 50, 25, 12.5, and 6.25 μg mL^−1^) of the compounds as the horizontal coordinate (x) and the corresponding peak area as the vertical coordinate (y). The regression equations of compounds **1**–**3** and **5** were y = 149.58x + 77.93 (*R*^2^ = 0.9965), y = 118.66x − 136.21 (*R*^2^ = 0.9980), y = 174.9x + 72.05 (*R*^2^ = 0.9994), and y = 90.255x + 297.19 (*R*^2^ = 0.9936), respectively. The quantitative analyses were repeated three times, and the average concentrations of the allelochemicals were calculated.

## 4. Conclusions

In summary, *L. perenne* could effectively inhibit the aboveground height and biomass of *A. adenophora* in the growth competition assay. In addition, seven norsesquiterpenes (**1**–**7**) were isolated and identified from the whole plant of *L. perenne*, and most of the compounds exhibited potent allelopathic effects on root length, stem length, and seed germination of *A. adenophora* and *L. sativa*, especially compound **3**. Moreover, active compounds were released into the environment through root secretion and rainwater leaching to negatively affect the growth of *A. adenophora.* Furthermore, the release contents of compounds **1** and **3** showed a gradually increasing trend with the increase in time. Our findings not only helped reveal the mechanism of *L. perenne*’s replacement control of *A. adenophora* from the perspective of allelopathy but also opened up new ways for the exploitation and utilization of norsesquiterpenes from *L. perenne*.

## Data Availability

All details and data can be found in the text.

## Supplementary Materials

The following supporting information can be downloaded at https://www.mdpi.com/article/10.3390/molecules30112384/s1. Figures S1−S14: NMR spectrum of compounds **1**–**7**. Figure S15: The isolation flowchart of norsesquiterpenes from *L. perenne*. Figure S16: Allelopathic effects of water extract of *L. perenne* on *A. adenophora*.

## References

[1] Bonnamour A., Gippet J.M.W., Bertelsmeier C., Gurevitch J. (2021). Insect and plant invasions follow two waves of globalisation. Ecol. Lett..

[2] Seebens H., Blackburn T.M., Dyer E.E., Genovesi P., Hulme P.E., Jeschke J.M., Pagad S., Pysek P., Winter M., Arianoutsou M. (2017). No saturation in the accumulation of alien species worldwide. Nat. Commun..

[3] Sobuj N., Byun C. (2023). A synthesis of plant invasion control: Important factors to consider when choosing a control method. Écoscience.

[4] Weidlich E.W.A., Flórido F.G., Sorrini T.B., Brancalion P.H.S. (2020). Controlling invasive plant species in ecological restoration: A global review. J. Appl. Ecol..

[5] Piemeisel R.L., Carsner E. (1951). Replacement control and biological control. Science.

[6] Li W., Luo J., Tian X., Chow W.S., Sun Z., Zhang T., Peng S., Peng C. (2015). A new strategy for controlling invasive weeds: Selecting valuable native plants to defeat them. Sci. Rep..

[7] Jia P., Wang J., Liang H., Wu Z.H., Li F., Li W. (2022). Replacement control of *Mikania micrantha* in orchards and its eco-physiological mechanism. Front. Ecol. Evol..

[8] Chen B.M., Liao H.X., Chen W.B., Wei H.J., Peng S.L. (2017). Role of allelopathy in plant invasion and control of invasive plants. Allelopathy J..

[9] Hierro J.L., Callaway R.M. (2021). The ecological importance of allelopathy. Annu. Rev. Ecol. Evol. Syst..

[10] Khamare Y., Chen J., Marble S.C. (2022). Allelopathy and its application as a weed management tool: A review. Front. Plant Sci..

[11] Putnam A.R. (1988). Allelochemicals from plants as herbicides. Weed Technol..

[12] Wang X., Yang X.J., Fu H.Y., He W., Wang Y.X., Sampietro D.A., Yang S.X., Kuang Y. (2022). Herbicide potential of new phytotoxins structurally based on plant allelochemicals. Allelopathy J..

[13] Jayasinghe C., Badenhorst P., Jacobs J., Spangenberg G., Smith K. (2021). Image-based high-throughput phenotyping for the estimation of persistence of perennial ryegrass (*Lolium perenne* L.)—A review. Grass Forage Sci..

[14] Wims C.M., Delaby L., Boland T.M., O’Donovan M. (2014). Effect of pre-grazing herbage mass on dairy cow performance, grass dry matter production and output from perennial ryegrass (*Lolium perenne* L.) pastures. Animal.

[15] Dineen M., McCarthy B., Ross D., Ortega A., Dillon P., van Amburgh M.E. (2021). Characterization of the nutritive value of perennial ryegrass (*Lolium perenne* L.) dominated pastures using updated chemical methods with application for the Cornell Net Carbohydrate and Protein System. Anim. Feed Sci. Technol..

[16] Zhang B., Zhang H., Jing Q., Wang J. (2020). Light pollution on the growth, physiology and chlorophyll fluorescence response of landscape plant perennial ryegrass (*Lolium perenne* L.). Ecol. Indic..

[17] Poudel A.S., Jha P.K., Shrestha B.B., Muniappan R. (2019). Biology and management of the invasive weed *Ageratina adenophora* (Asteraceae): Current state of knowledge and future research needs. Weed Res..

[18] Kluge R.L. (1991). Biological control of crofton weed, *Ageratina adenophora* (Asteraceae), in South Africa. Agr. Ecosyst. Environ..

[19] Yuan C., Wang Q., Chen Y., Zhang L.D., Tan L., Fu R.H., Yang J.T., Li Y., Liu M., Compton S.G. (2021). Impacts of a biocontrol agent on invasive *Ageratina adenophora* in Southwest China: Friend or foe?. Biol. Control.

[20] Ren Z., Okyere S.K., Wen J., Xie L., Cui Y., Wang S., Wang J., Cao S., Shen L., Ma X. (2021). An overview: The toxicity of *Ageratina adenophora* on animals and its possible interventions. Int. J. Mol. Sci..

[21] Zhao L., Li B.P., Meng L., Zhu H.W. (2008). The relative competitive ability of perennial *Lolium perenne* and the invasive alien weed, *Eupatorium adenophorum* (Compositae) at different nitrogen and phosphorus levels in the seedling stage. Acta Prataculturae Sin..

[22] Zhu H., Meng L., Li B. (2007). Relative competitive ability of *Lolium perenne* and the invasive alien weed, *Eupatorium adenophorum* (Compositae) at seedling stage. Chin. J. Appl. Environ. Biol..

[23] Matsuo T., Bongers F., Martínez-Ramos M., van der Sande M.T., Poorter L. (2024). Height growth and biomass partitioning during secondary succession differ among forest light strata and successional guilds in a tropical rainforest. Oikos.

[24] Kim K.H., Lee K.H., Choi S.U., Kim Y.H., Lee K.R. (2008). Terpene and phenolic constituents of *Lactuca indica* L. Arch. Pharm. Res..

[25] Park J.H., Lee D.G., Yeon S.W., Kwon H.S., Ko J.H., Shin D.J., Park H.S., Kim Y.S., Bang M.H., Baek N.I. (2011). Isolation of megastigmane sesquiterpenes from the Silkworm (*Bombyx mori* L.) droppings and their promotion activity on HO-1 and SIRT1. Arch. Pharm. Res..

[26] Yan Z.H., Han Z.Z., Hu X.Q., Liu Q.X., Zhang W.D., Liu R.H., Li H.L. (2013). Chemical constituents of *Euonymus alatus*. Chem. Nat. Compd..

[27] Le H.L., Nguyen T.M.H., Vu T.T., Nguyen T.T.O., Ly D.T., Le N.T., Nguyen V.H., Nguyen T.V.A. (2022). Potent antiplatelet aggregation, anticoagulant and antioxidant activity of aerial Canna x generalis L.H Bailey & E.Z Bailey and its phytoconstituents. S. Afr. J. Bot..

[28] Yuan Z., Zheng X., Zhao Y., Liu Y., Zhou S., Wei C., Hu Y., Shao H. (2018). Phytotoxic compounds isolated from leaves of the invasive weed *Xanthium spinosum*. Molecules.

[29] Morais A.M.M.B., Kumla D., Martins V.F.R., Alves A., Gales L., Silva A.M.S., Costa P.M., Mistry S., Kijjoa A., Morais R.M.S.C. (2024). Monoterpene hydroxy lactones isolated from *Thalassiosira* sp. microalga and their antibacterial and antioxidant activities. Molecules.

[30] Finch S.C., Prinsep M.R., Popay A.J., Wilkins A.L., Webb N.G., Bhattarai S., Jensen J.G., Hawkes A.D., Babu J.V., Tapper B.A. (2020). Identification and structure elucidation of epoxyjanthitrems from *Lolium perenne* infected with the endophytic fungus *Epichloë festucae* var. lolii and determination of the tremorgenic and anti-insect activity of Epoxyjanthitrem I. Toxins.

[31] Reddy P., Deseo M.A., Ezernieks V., Guthridge K., Spangenberg G., Rochfort S. (2019). Toxic indole diterpenes from endophyte-infected perennial ryegrass *Lolium perenne* L.: Isolation and stability. Toxins.

[32] Hossen K., Asato Y., Teruya T., Kato-Noguchi H. (2023). Identification of four allelopathic compounds including a novel compound from *Elaeocarpus foribundus* Blume and determination of their allelopathic activity. J. Environ. Manag..

[33] Macias F.A., Varela R.M., Torres A., Oliva R.M., Molinillo J.M.G. (1998). Bioactive norsesquiterpenes from *Helianthus annuus* with potential allelopathic activity. Phytochemistry.

[34] Kyaw E.H., Iwasaki A., Suenaga K., Kato-Noguchi H. (2022). Allelopathy of the medicinal plant *Dregea volubilis* (L.f.) Benth.ex Hook.f. and its phytotoxic substances with allelopathic activity. Agronomy.

[35] Ma W., Tang S., Deng Z., Zhang D., Zhang T., Ma X. (2022). Root exudates contribute to belowground ecosystem hotspots: A review. Front. Microbiol..

[36] Wang A.K., Huang K.W., Ning Y.L., Bi Y.F. (2024). Allelochemicals from *Moso bamboo*: Identification and their effects on neighbor species. Forests.

