# Supplementary material for: Norsesquiterpenes from Lolium perenne and Their Replacement Control of an Invasive Plant, Ageratina adenophora, Through Allelopathy
Source: Molecules. 2025 May 29;30(11):2384. doi: 10.3390/molecules30112384 (PMC12156316; doi:10.3390/molecules30112384)

Supporting Information

Norsesquiterpenes from *Lolium perenne* and their replacement control of an invasive plant *Ageratina adenophora* though allelopathy

Wenbo Shi [a,1], Tong An [a,1], Xiaomin Yang [1], Youlin Li [2], Amanula Yimingniyazi [3], Zhixiang Liu [1,*], Yulong Feng [1,*]

[1] College of Bioscience and Biotechnology, Shenyang Agricultural University, Shenyang 110866, P.R. China

[2] College of Resources and Environment, Yunnan Agricultural University, Kunming 650500, P.R. China

[3] Xinjiang Key Laboratory for Ecological Adaptation and Evolution of Extreme Environment Biology, College of Life Sciences, Xinjiang Agricultural University, Urumqi 830052, P.R. China

[a] The authors contributed equally to this work.

*Corresponding authors. Phone: +86-24-88487163. Fax: +86-24-88492799.

E-mail addresses: liuzhixiang327@163.com (Z.-X. Liu); fyl@syau.edu.cn (Y.-L. Feng)

Figure S1 The ^{1}H-NMR spectrum of compound **1**

Figure S2 The ^{13}C-NMR spectrum of compound **1**

Figure S3 The ^{1}H-NMR spectrum of compound **2**

Figure S4 The ^{13}C-NMR spectrum of compound **2**

Figure S7 The ^{1}H-NMR spectrum of compound **4**

Figure S8 The ^{13}C-NMR spectrum of compound **4**

Figure S9 The ^{1}H-NMR spectrum of compound **5**

Figure S10 The ^{13}C-NMR spectrum of compound **5**

Figure S11 The ^{1}H-NMR spectrum of compound **6**

Figure S12 The ^{13}C-NMR spectrum of compound **6**

Figure S13 The ^{1}H-NMR spectrum of compound **7**

Figure S14 The ^{13}C-NMR spectrum of compound **7**

Figure S15 The isolation flowchart of norsesquiterpenes from *L. perenne*

Figure S16 Allelopathic effects of water extract of *L. perenne* on root length, stem length and seed germination of *A. adenophora*. Different letters indicated significant differences between different growth indicators for each compound ($p < 0.05$).

Fig. S1 The ^{1}H-NMR spectrum of compound **1**

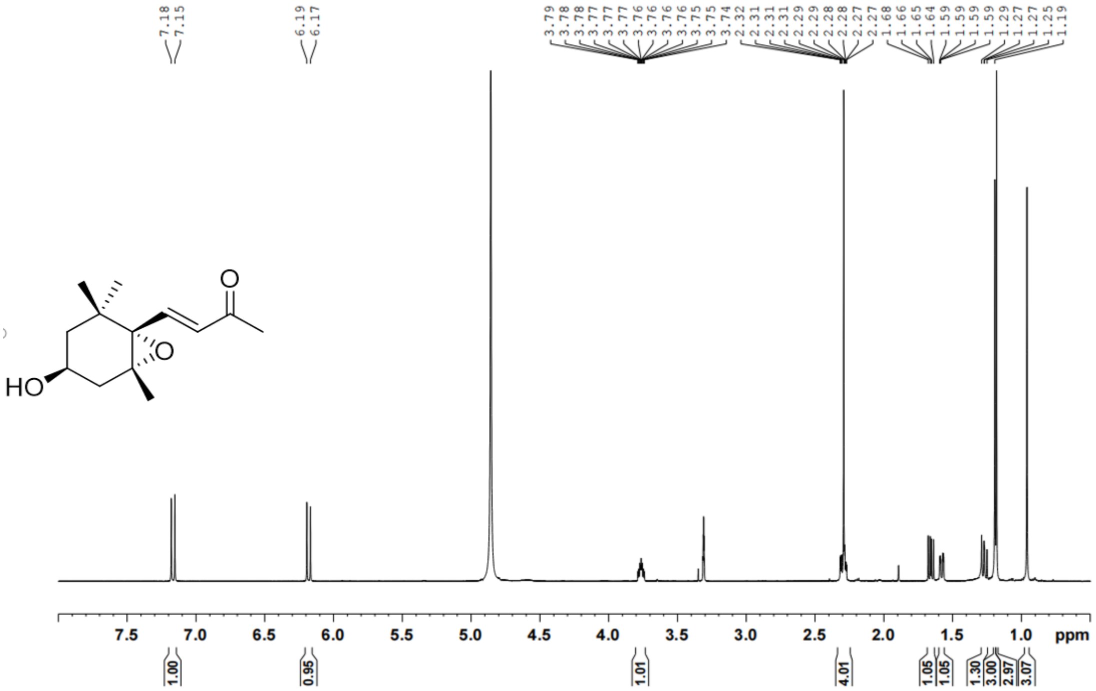

Fig. S2 The ^{13}C-NMR spectrum of compound **1**

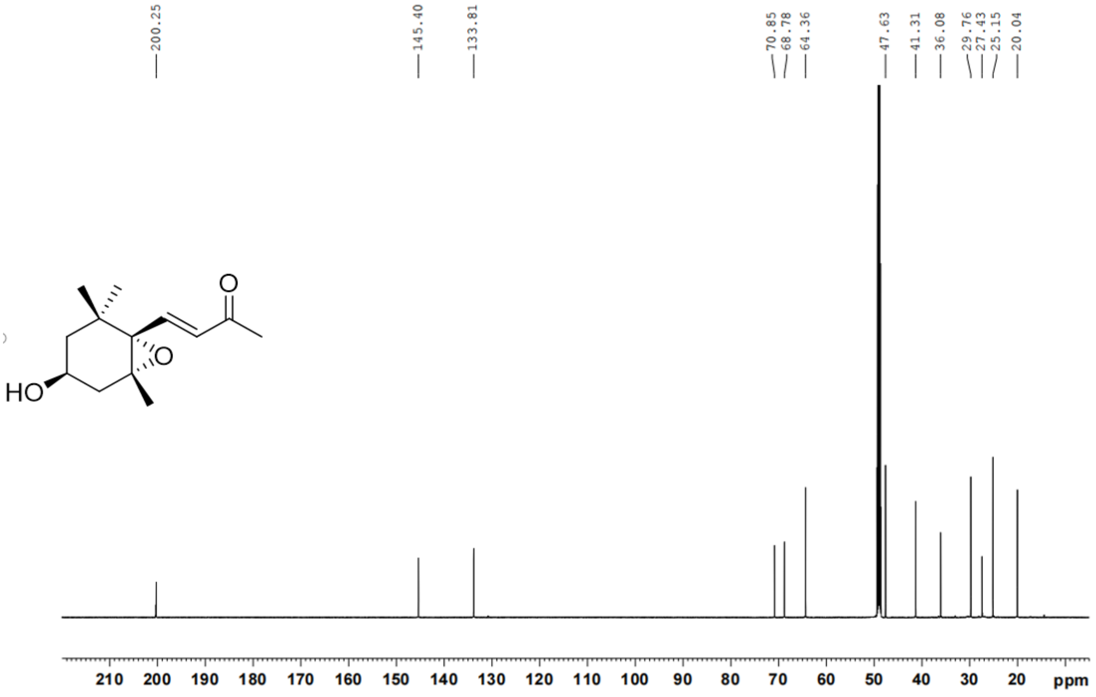

Fig. S3 The ^{1}H-NMR spectrum of compound **2**

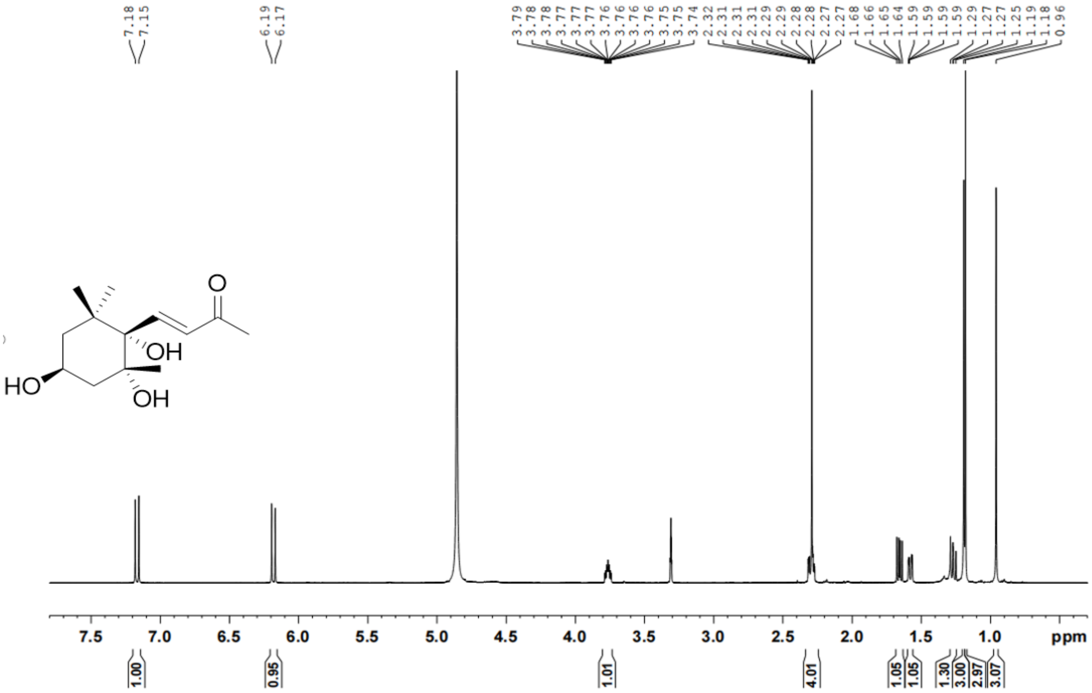

Fig. S4 The ^{13}C-NMR spectrum of compound **2**

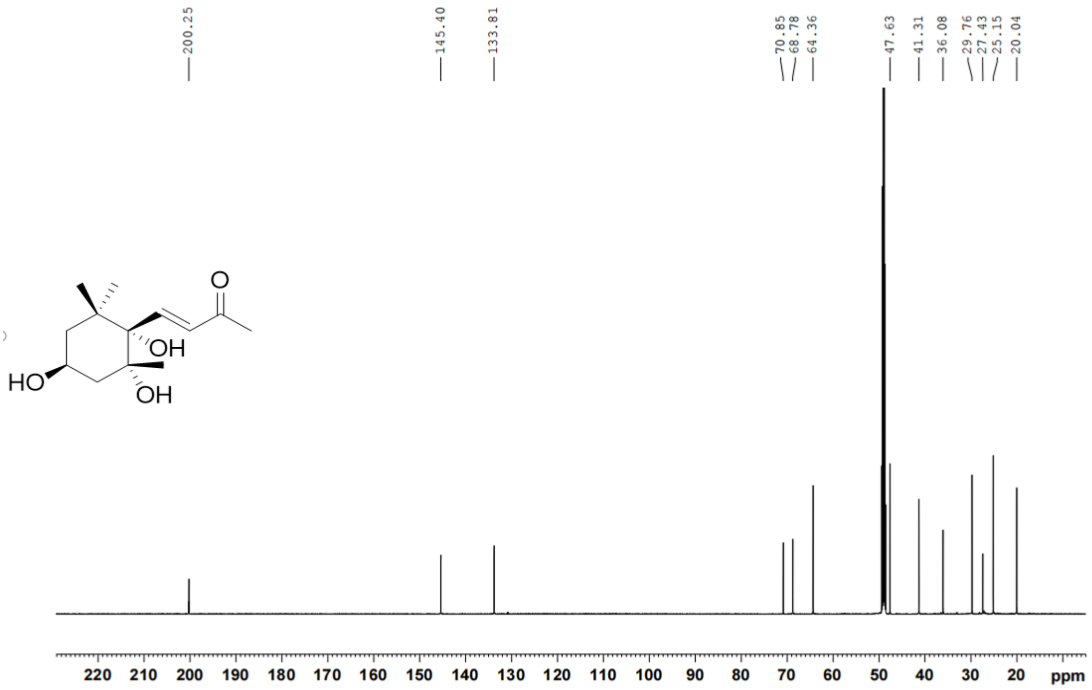

Fig. S5 The ^{1}H-NMR spectrum of compound **3**

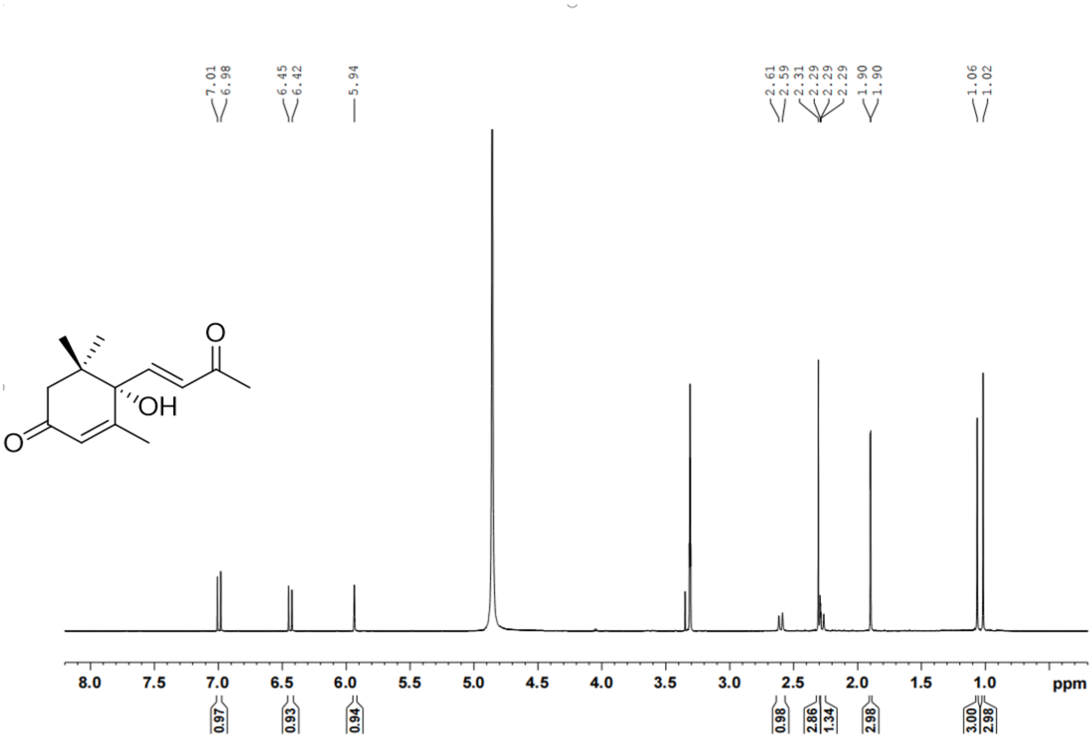

Fig. S6 The ^{13}C-NMR spectrum of compound **3**

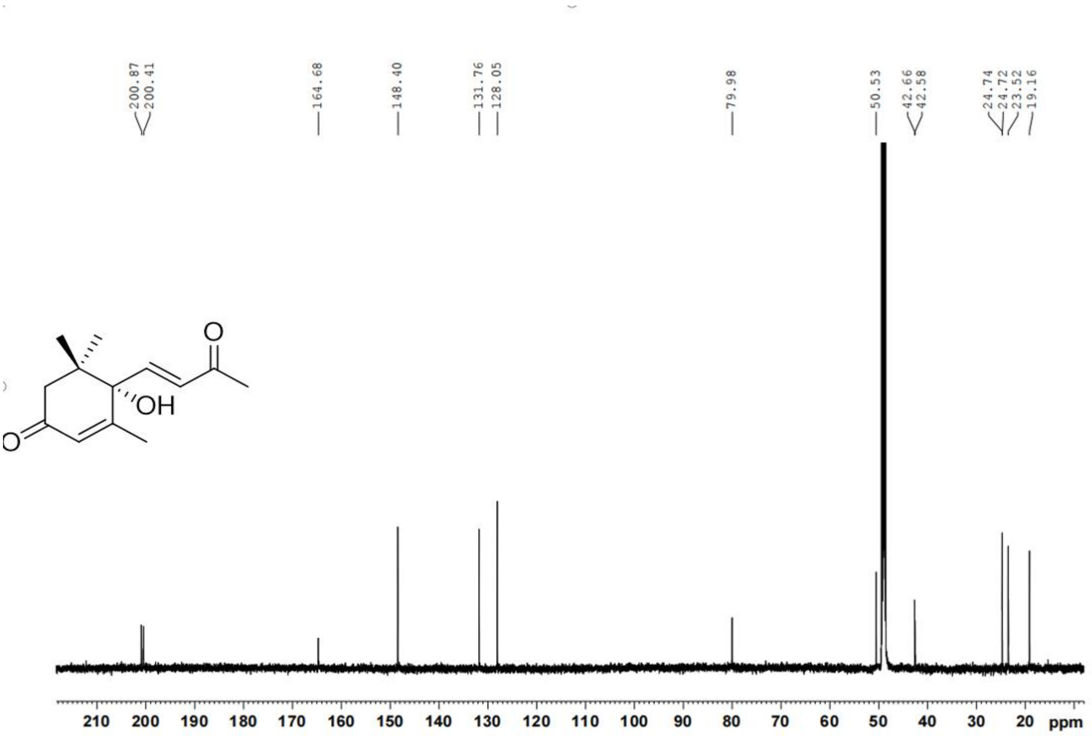

Fig. S7 The ^{1}H-NMR spectrum of compound **4**

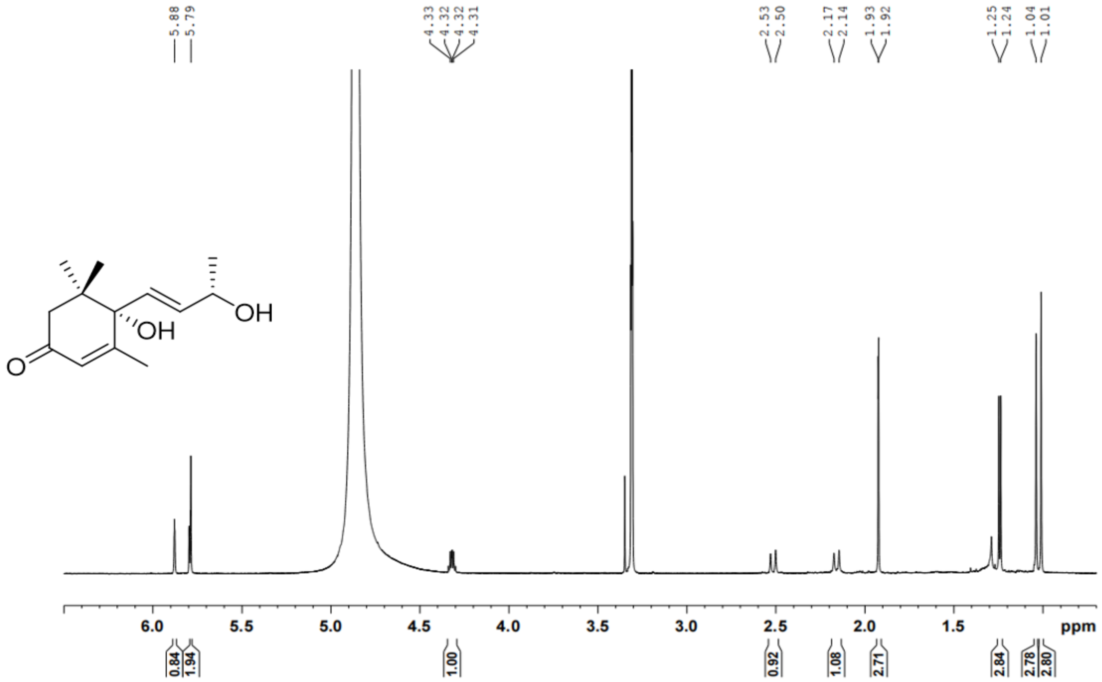

Fig. S8 The ^{13}C-NMR spectrum of compound **4**

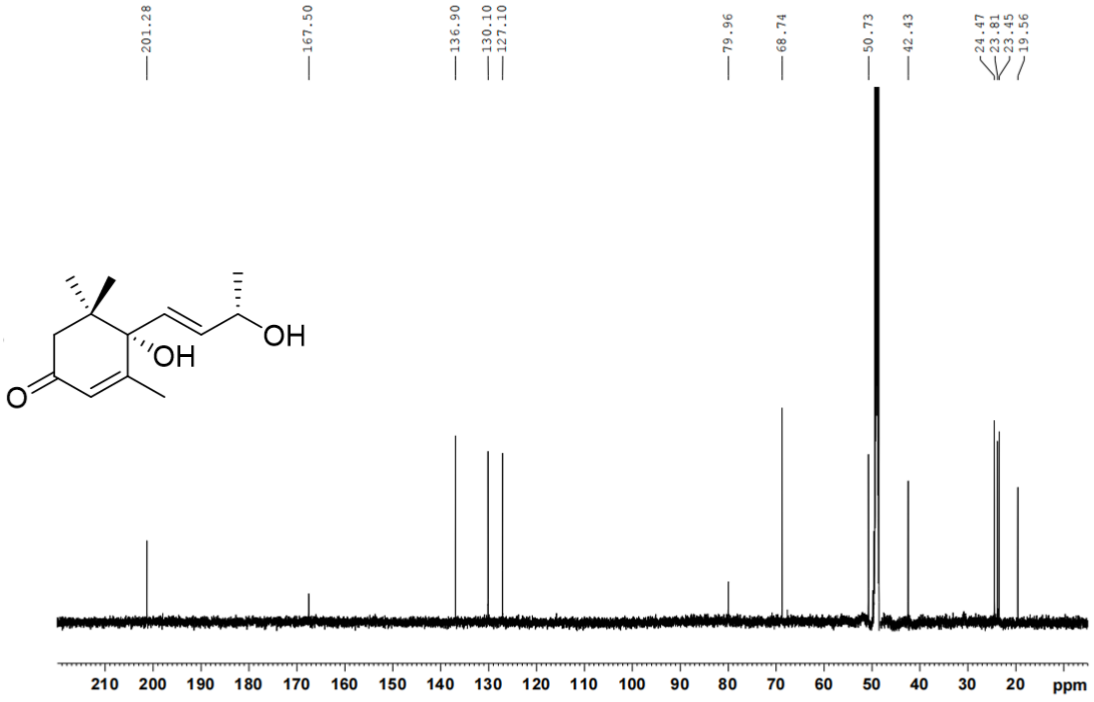

Fig. S9 The ^{1}H-NMR spectrum of compound **5**

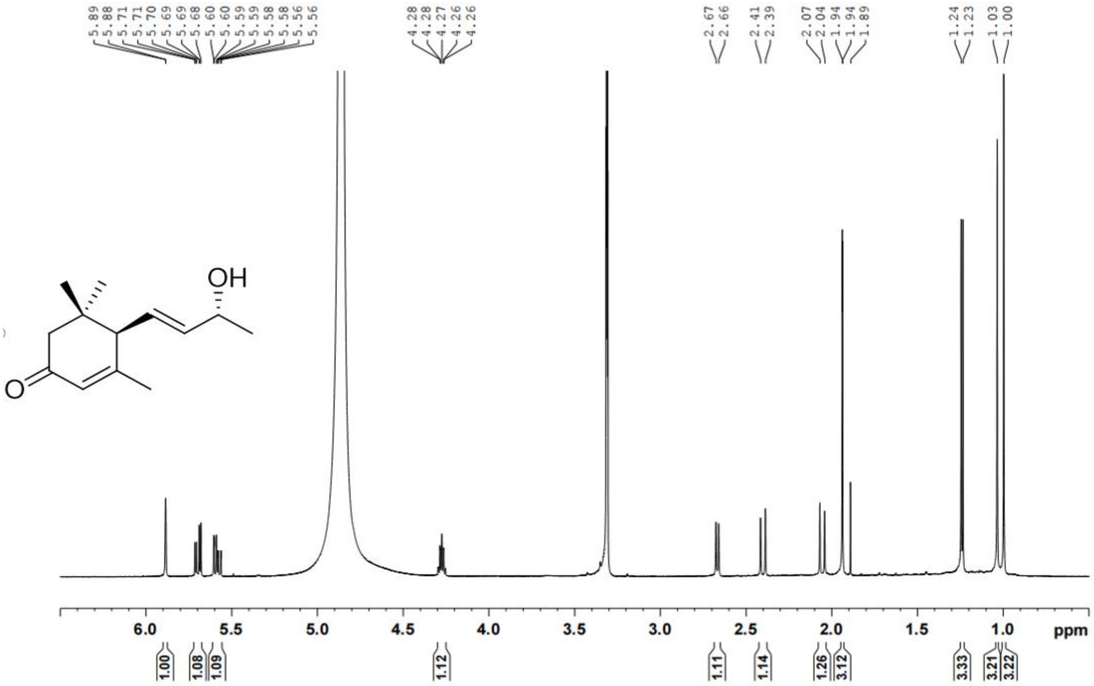

Fig. S10 The ^{13}C-NMR spectrum of compound **5**

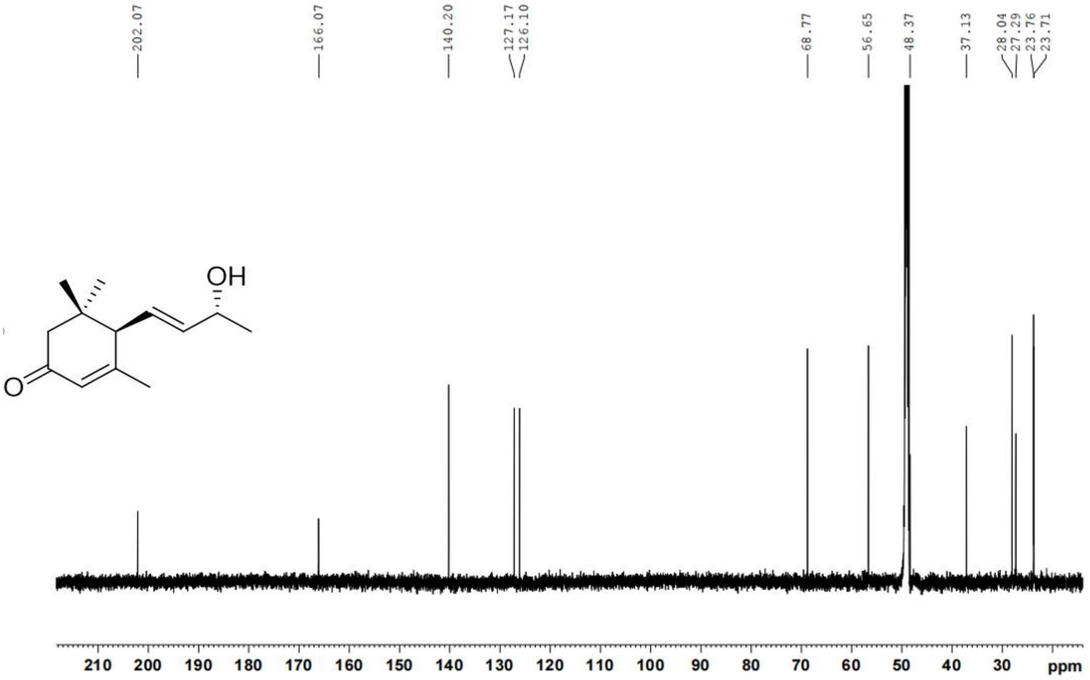

Fig. S11 The ^{1}H-NMR spectrum of compound **6**

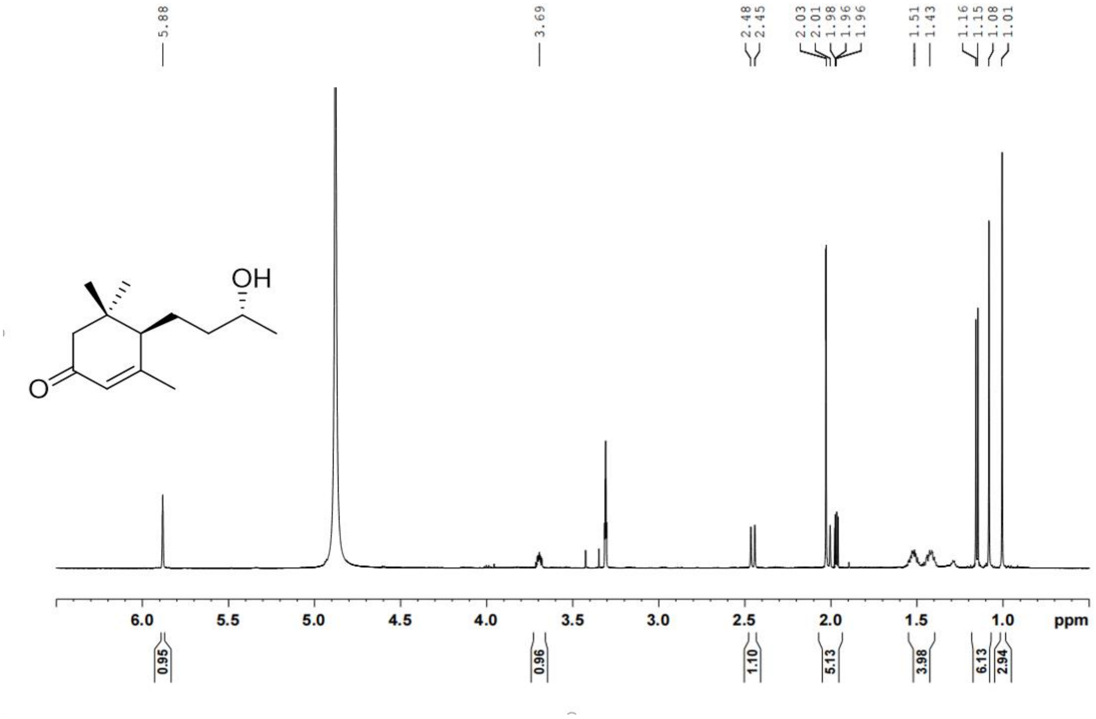

Fig. S12 The ^{13}C-NMR spectrum of compound **6**

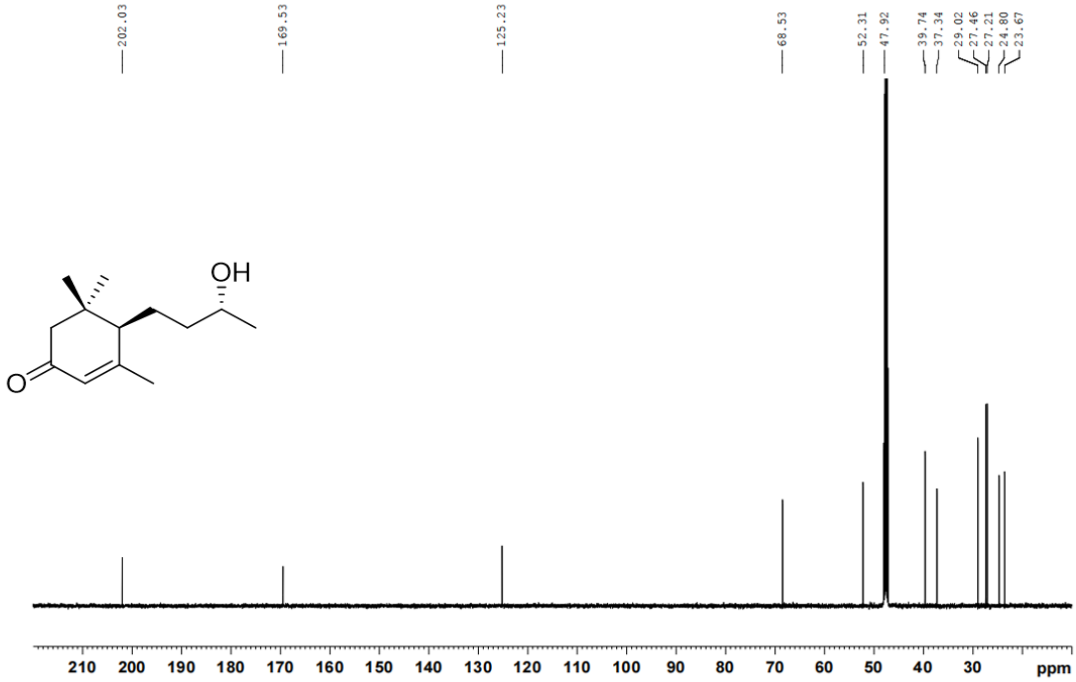

Fig. S13 The ^{1}H-NMR spectrum of compound **7**

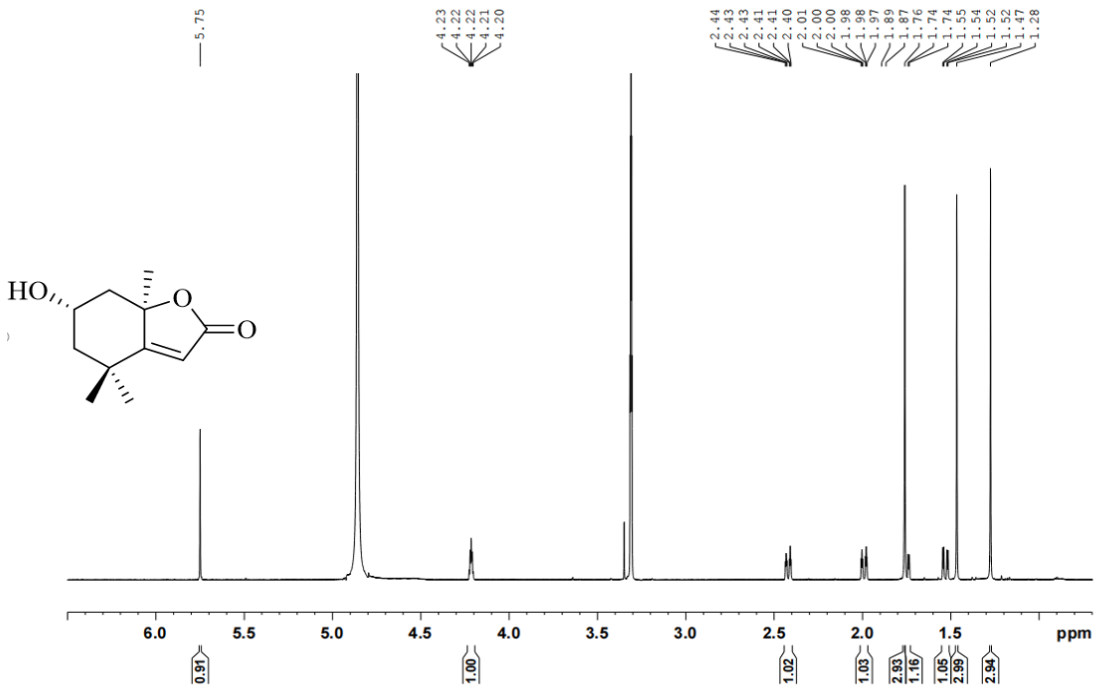

Fig. S14 The ^{13}C-NMR spectrum of compound **7**

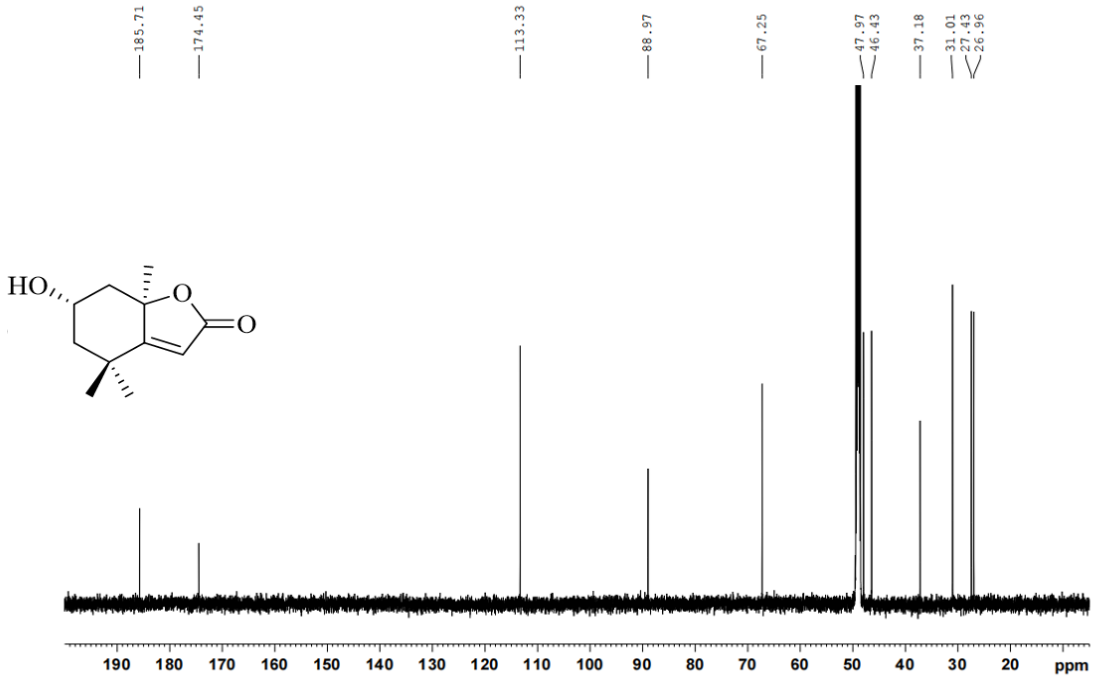

Fig. S15 The isolation flowchart of norsesquiterpenes from *L. perenne*

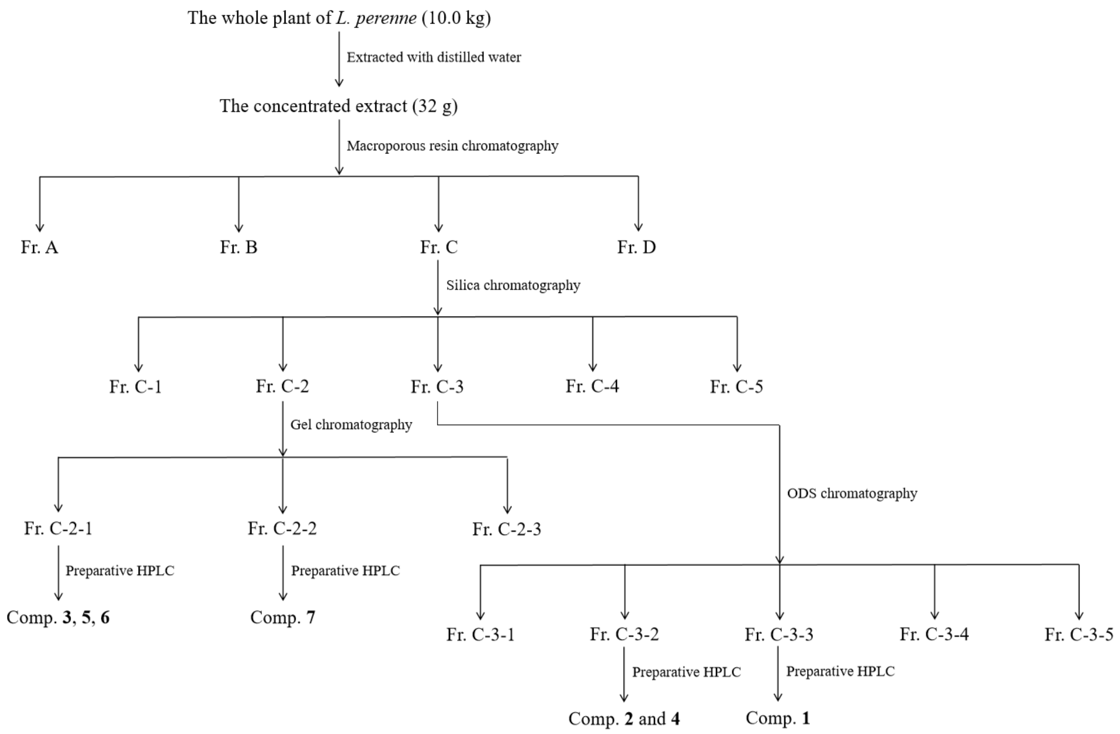

Fig. S16 Allelopathic effects of water extract of *L. perenne* on root length, stem length and seed germination of *A. Adenophora*. Different letters indicated significant differences between different growth indicators for each compound ($p < 0.05$).

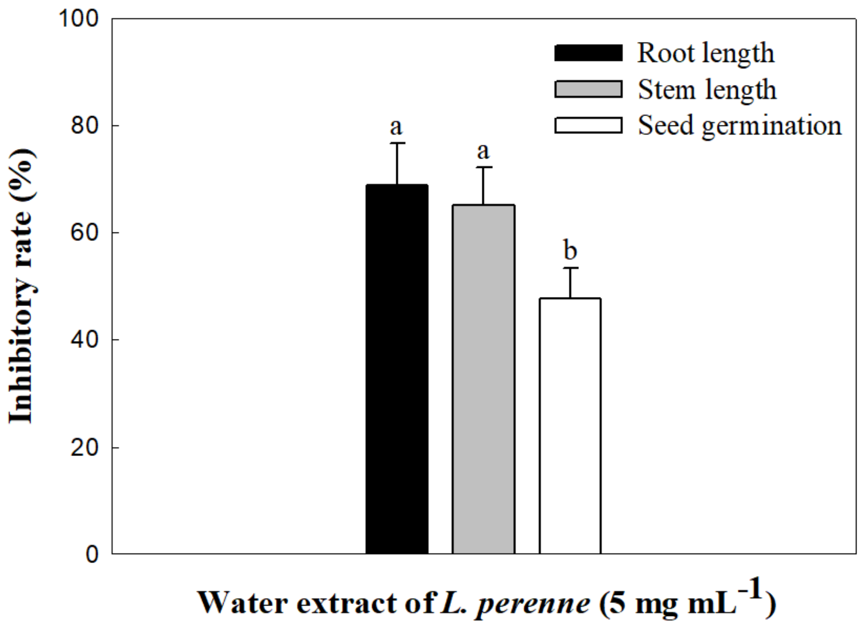

Supplement: Supplementary file 1 [file molecules-30-02384-s001.zip › molecules-3621567-supplementary.pdf]
